# Reliability and Validity of the Chinese Version of the Children’s Depression Rating Scale—Revised (CDRS-R)

**DOI:** 10.3390/healthcare13070734

**Published:** 2025-03-26

**Authors:** Yajie Huang, Xuemei Li, Jie Li, Tingting Lei, Yuqian He, Wenjing Wang, Xiaoxia Xu, Yao Li, Xinyu Zhou

**Affiliations:** Department of Psychiatry, Key Laboratory of Major Brain Disease and Aging Research (Ministry of Education), The First Affiliated Hospital of Chongqing Medical University, Chongqing 400016, China; yajiehuanghy@126.com (Y.H.); xuemeililucky@126.com (X.L.); lijiedada123@163.com (J.L.); annalaytt@163.com (T.L.); hhhyuq2022@163.com (Y.H.); nutshellx@163.com (W.W.); latesummer777@163.com (X.X.); dogegg2025@163.com (Y.L.)

**Keywords:** major depressive disorder, adolescents, CDRS-R, reliability and validity, factor structures

## Abstract

**Background/Objectives:** The Children’s Depression Rating Scale—Revised (CDRS-R) is a well-established tool to evaluate depressive symptoms in adolescents, yet its psychometric properties in China have not been thoroughly validated. The present research aimed to assess the Chinese version of the CDRS-R in adolescents from China. **Methods:** This study included 360 adolescents: 180 were diagnosed with major depressive disorder (MDD) and 180 were healthy controls (HCs). Internal consistency, convergent validity, and factor structure were evaluated, while receiver operating characteristic (ROC) analysis was employed to establish cutoff scores. **Results:** The Chinese CDRS-R demonstrated high internal reliability (Cronbach’s α = 0.966) and strong correlations with related measures, confirming its convergent validity. Confirmatory factor analysis supported the original four-factor structure. ROC analysis indicated that the optimal cutoff score for diagnosing MDD was 48, effectively distinguishing MDD from HCs. **Conclusions:** The findings confirm that the Chinese CDRS-R is a reliable instrument for assessing depressive symptoms in Chinese adolescents, making it suitable for both clinical and research purposes.

## 1. Introduction

Major depressive disorder (MDD) is a prevalent mental health condition that affects individuals globally and is a significant contributor to the global disease burden [1]. During adolescence, there is a significant rise in the prevalence of MDD [2]. An epidemiological study conducted in China in 2021 on mental disorders in children and adolescents revealed that the rate of MDD prevalence was 2.0% [3]. It is worth noting that suicide ranks as the second or third leading factor contributing to mortality in adolescents, with depressed adolescents demonstrating a higher inclination toward suicidal ideation compared to their non-depressed counterparts [4]. This underscores the imperative for prompt identification and intervention in cases of adolescent depression. However, recognizing depressive disorders in adolescents may pose difficulties in clinical settings due to their frequent mood fluctuations, irritability, and symptom fluctuation. In addition, depression may be overlooked when primary complaints involve unexplained physical symptoms, other psychiatric symptoms, school refusal, or behavioral problems [5].

In clinical practice, medical professionals commonly rely on specific instruments to assess the severity of psychiatric disorders, including depression. Several self-report instruments for MDD have been used to assess the depressive symptoms of patients in China: for instance, the Beck Depression Inventory (BDI) and the Center for Epidemiologic Studies Depression Scale for Children (CES-DC) [6,7,8,9]. Currently, there is a lack of interviewer rating scales tailored specifically for assessing adolescent depression in China. Therefore, the Hamilton Depression Rating Scale (HAMD) is frequently used in China to assess adolescents with depression [10,11]. However, the HAMD presents several weaknesses. Firstly, it was primarily developed for adults, and its content validity may not fully capture the key clinical characteristics of depression in adolescents [12]. Secondly, the HAMD is typically rated solely by clinicians, thus limiting the acquisition of crucial information from other sources (individuals familiar with the patient). Thirdly, the numerous versions of the HAMD present significant challenges in selection for clinicians [13].

The Children’s Depression Rating Scale—Revised (CDRS-R) is a brief assessment tool that involves a semi-structured interview with the child or a close adult [14]. Administrable within 15-to-20 min, the scale is primarily designed for children aged 6-to-12 but remains applicable to adolescents [15]. Adolescents complete the initial 14 items while an observer rates the child’s nonverbal conduct for items 15-to-17. Unlike self-report inventories, a direct interview facilitates engagement with isolated and withdrawn adolescents, with isolation and withdrawal being common among those with depression, thereby enabling a more accurate assessment. Numerous studies have examined the psychometric properties of different language versions of the CDRS-R [16,17]. However, previous analyses focusing exclusively on structural validity have employed exploratory factor analysis, resulting in variations in both the number and content of the factors. Consequently, significant gaps persist in the literature concerning validation factor analysis of the CDRS-R, necessitating comprehensive validation studies to ascertain the scale’s factor structure and ensure its psychometric integrity across diverse linguistic and cultural contexts [18].

Presently, the CDRS-R is the most widely utilized tool for evaluating the intensity of depression in children and adolescents. It is also extensively utilized in global clinical studies to monitor variations in depressive symptoms [18]. Although the CDRS-R is widely used in clinical research, no studies have investigated the psychometric properties of its Chinese version. This research evaluated the reliability and validity of the Chinese version of the CDRS-R and examined its applicability in Chinese adolescents. The goal is to establish a valid and reliable tool for assessing depressive symptoms in this population and provide a valuable reference for future large-scale studies on MDD in China.

## 2. Methods

### 2.1. Participants

Participants were recruited from the First Affiliated Hospital of Chongqing Medical University. The inclusion criteria for the MDD group (*n* = 180) were as follows: (1) aged 12–18 years; (2) a diagnosis of MDD confirmed using the Chinese version of the Kiddie Schedule for Affective Disorders and Schizophrenia—Present and Lifetime Version (K-SADS-PL-C) [19], based on the Diagnostic and Statistical Manual of Mental Disorders—Fifth Edition (DSM-5) criteria, with clinically significant depressive symptoms verified by a psychiatrist; and (3) proficient in Chinese (able to understand, speak, read, and write). The inclusion criteria for healthy controls (*n* = 180) were as follows: (1) aged 12–18 years; (2) no current psychiatric illness and history of mood disorder (according to the K-SADS-PL-C DSM-5); and (3) absence of physical illness and no medication use (confirmed by a psychiatrist).

The exclusion criteria included the following: (1) presence or history of serious medical, neurological, or psychiatric illnesses (MDD had to be the primary cause for dysfunction, although patients with concurrent disorders, such as anxiety, were included); (2) alcohol or substance abuse; (3) first-degree relatives with bipolar I disorder; and (4) lactating or pregnant women.

Informed consent, obtained in writing from the participants and their guardians, followed a comprehensive explanation of the research details. This study obtained ethical clearance from the Medical Ethics Committee of the First Affiliated Hospital of Chongqing Medical University (Number: 2023-009-01).

### 2.2. Sample Size

In confirmatory factor analysis, it is recommended to have at least 10 respondents for each freely estimated parameter, following the rule of 10 [20]. In this study, we had sufficient data for the number of factors (latent variables) in the CDRS-R, with a sample size of 360.

### 2.3. Scale Translation

Permission was obtained from the copyright owner (Western Psychological Services). The method for obtaining the copyright of CDRS-R is provided in the Supplementary Materials. The Simplified Chinese translation of the original English CDRS-R followed Brislin’s method of forward and backward translation [21]. Initially, an English version was translated into Chinese by a psychiatrist with a master’s degree, who was proficient in both languages and well-versed in psychiatric terminology. Subsequently, the translated version underwent rigorous scrutiny and refinement by a clinical psychiatry professor to ensure conceptual equivalence and suitability in the Chinese context. To verify the fidelity of the translation, another bilingual psychiatrist independently translated the Chinese version back into English, without prior knowledge of the original scale’s content. This step aimed to identify any discrepancies or ambiguities in the translation, which were addressed through collaborative efforts between the translators and clinical experts. The final Chinese version of the scale underwent thorough evaluation and endorsement by two experienced professors in the field, affirming its accuracy and clarity. Crucially, the translation retained all 17 items from the original scale, maintaining its integrity and comprehensiveness in assessing depressive symptoms among Chinese-speaking adolescents.

### 2.4. Measures

#### 2.4.1. K-SADS-PL-C DSM-5

The K-SADS-PL DSM-5 is an assessment tool designed to evaluate psychiatric disorders in youth between the ages of 6 and 18, providing a comprehensive mental health evaluation [22]. Yue Dun et al. translated the Chinese version of the K-SADS-PL DSM-5 and reported fair-to-excellent inter-rater reliability (0.537–1.000) and test–retest reliability (0.468–0.885) for affective disorder. The convergent validity between affective disorder and neurodevelopment disorder was considered good [19].

Consistent with previous studies, our analysis explored the relationship between the CDRS-R and the depression subscale scores from the K-SADS-PL-C, with the subscale score derived from the sum of the following symptom ratings. These included mood disturbances, excessive guilt, and lack of interest. Fatigue, concentration difficulties, and psychomotor changes—such as agitation and retardation—were also considered. Sleep disturbances, including insomnia and hypersomnia, and changes in appetite, either increased or decreased, were included in the assessment. Suicidal ideation was also factored into the overall score [23,24].

#### 2.4.2. Children’s Depression Rating Scale—Revised (CDRS-R)

The CDRS-R comprises 17 questions, yielding total scores ranging from 17 to 113. For the first 14 items of the CDRS-R, ratings were based on the adolescent’s verbal responses, with input from parents when necessary. Adolescents typically provide more accurate reports of symptoms such as sleep disturbances, excessive fatigue, depressed mood, and morbid thoughts, while parents tend to offer more reliable reports for symptoms related to impaired school performance and social withdrawal [25]. For the last three items—depressed affect, listless speech, and hypoactivity—trained psychiatrists assessed the adolescent’s nonverbal behaviors during the interview. A total score of 40 or higher indicated clinically significant depressive symptoms [14].

#### 2.4.3. Beck Depression Inventory (BDI)

The BDI is a self-report inventory consisting of 21 items used to evaluate common depression-related symptoms and attitudes. Depression severity was divided into six levels: 0–10 (normal), 11–16 (mild disturbance), 17–20 (borderline depression), 21–30 (moderate depression), 31–40 (severe depression), and over 40 (extreme depression) [6]. The Chinese BDI showed an acceptable psychometric reliability [7].

#### 2.4.4. Clinical Global Impressions—Severity (CGI-S)

The CGI-S is a 7-point scale with only one entry to assess overall impressions of severity, rather than individual depressive symptoms. Clinicians rate the severity of the patient’s illness from a global perspective relative to their experience with MDD. It serves as a practical, easily administered measurement tool in busy clinical practice settings [26]. As reported by Kim et al., a strong correlation of 0.84 was observed between the scores of the CDRS-R and CGI-S [24].

### 2.5. Procedure

The present study used a cross-sectional design. Before conducting interviews, interviewers received training on clinician rating scales, diagnostic classifications, and critical differential diagnoses in child psychiatry. Inter-rater reliability was validated during this training period. All participants underwent assessments using the K-SADS-PL-C DSM-5, CDRS-R, BDI, and CGI-S.

### 2.6. Statistical Analyses

Data analysis was performed using SPSS version 26.0 and Mplus version 8 software. Descriptive statistics were computed to summarize sample characteristics. For continuous variables, means and standard deviations (SDs) were used. Frequencies and percentages were computed for categorical variables. These statistics provided an overview of the sample characteristics. The normality of the data was evaluated using the Kolmogorov–Smirnov and Shapiro–Wilk tests. As the data did not meet the assumption of normality, non-parametric tests were applied for analysis. The Mann–Whitney U test was utilized to assess differences in continuous variables between groups, while the Chi-square test was employed to evaluate the relationships among categorical variables. Effect sizes were determined to evaluate the extent of the differences noted among the groups. Based on Cohen’s guidelines, effect sizes fall into three categories: small (0.2–0.49), medium (0.5–0.79), and large (0.8 and above) [27].

Reliability was assessed using Cronbach’s alpha for CDRS-R, with a coefficient exceeding 0.7 indicating satisfactory internal consistency [28]. Spearman’s correlation coefficients were used to assess the associations between CDRS-R scores and those from the depression subscale of the K-SADS-PL-C DSM-5, as well as the BDI and CGI-S. Correlation analyses were conducted separately for participants with MDD and HCs to explore potential differences between the groups, and Fisher’s r-to-z transformation was applied to statistically compare correlation strengths. Spearman’s correlation coefficients of 0.10, 0.30, and 0.50 were interpreted as small, medium, and large, respectively [27].

Confirmatory factor analyses (CFAs) of the CDRS-R were conducted with Mplus version 8.0. The initial authors’ proposed four-factor models were subjected to testing [29]. The models were estimated using the Robust Maximum Likelihood (MLR) estimator. To assess model fit, the Chi-square statistic, comparative fit index (CFI), Tucker–Lewis index (TLI), standardized root mean square residual (SRMR), and root mean square error of approximation (RMSEA) were examined. A model was considered to have acceptable fit if the CFI exceeded 0.90, the TLI was greater than 0.90, the SRMR was less than 0.08, and the RMSEA was under 0.08 [30,31,32].

Receiver operating characteristic (ROC) analysis was used to measure the sensitivity, specificity, and area under the curve (AUC) for the CDRS-R. AUC values from 0.7 to 0.8, from 0.8 to 0.9, and >0.9 were indicative of acceptable, good, and excellent predictive ability, respectively [33]. The optimal cutoff for classification was identified by the point on the ROC curve closest to the upper left corner, balancing sensitivity and specificity [34].

## 3. Results

### 3.1. Characteristics of the Sample

The study sample consisted of 360 adolescents, with 180 (50%) diagnosed with MDD and 180 (50%) serving as healthy controls (HCs). The mean age for the MDD and HC groups was 14.72 (1.62) and 14.51 (1.17), respectively, with female proportions of 60% and 62.2%. No significant differences were found regarding age, BMI, or gender between the MDD and HC groups. Nevertheless, significant differences were noted in the K-SADS-PL-C depression subscale scores (*p* < 0.001, Cohen’s d = 4.01), CDRS-R scores (*p* < 0.001, Cohen’s d = 4.18), BDI scores (*p* < 0.001, Cohen’s d = 1.89), and CGI-S scores (*p* < 0.001, Cohen’s d = 4.35) (see Table 1).

### 3.2. Reliability

The CDRS-R demonstrated high internal consistency, as indicated by a Cronbach’s alpha value of 0.966. According to the original authors, the CDRS-R is structured around a four-factor model encompassing mood, somatic, subjective, and behavioral dimensions [29]. The lowest Cronbach’s alpha value for the four factors was 0.848, while the highest was 0.910. All 17 items showed significant correlations with the total CDRS-R score, ranging from 0.642 to 0.938 (see Table 2).

Additionally, the internal consistency of the CDRS-R was analyzed separately for MDD and HC groups. Cronbach’s alpha values were 0.837 for the MDD group and 0.874 for the HC group, indicating good internal consistency in both groups.

### 3.3. Validity Analysis

#### 3.3.1. Convergent Validity

The CDRS-R total scores were significantly correlated with the depression subscale scores from K-SADS-PL-C, BDI, and CGI-S (Table 3), indicating good convergent validity.

In both the MDD and HC groups, CDRS-R scores showed significant positive associations with these measures, demonstrating good convergent validity. The correlation results are presented in Supplementary Tables S1 and S2. Fisher’s Z-test was used to compare the differences in the correlations between CDRS-R and the K-SADS-PL-C depression subscale, BDI, and CGI-S across the MDD and HC groups. The relationship between the CDRS-R and the K-SADS-PL-C depression subscale was significantly stronger in the MDD group compared to the HC group (Z = 4.44, *p* < 0.001). In contrast, no significant differences were observed in the correlations with the BDI and CGI-S.

#### 3.3.2. Confirmatory Factor Analysis

**Figure 1:**
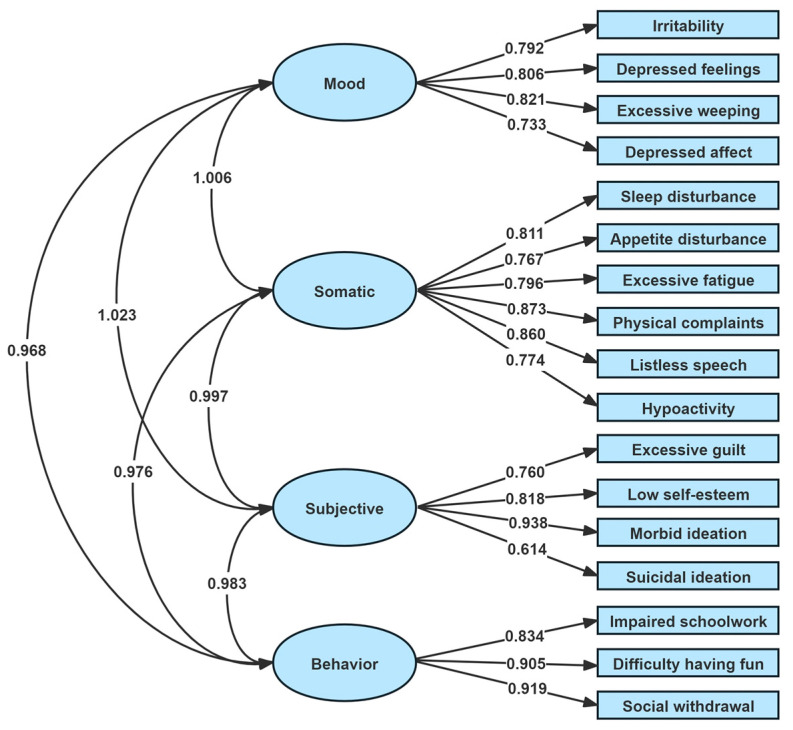
Standardized factor loadings of the four-factor model of the Children’s Depression Rating Scale—Revised (CDRS-R).

CFA was used to examine the factor structure of the CDRS-R based on the original model proposed by Poznanski [29]. The results of the model fit indices indicate a good fit to the four-factor model (TLI = 0.948, CFI = 0.957, RMSEA = 0.067 (90% CI: 0.058–0.077), SRMR = 0.049). Figure 1 presents the CFA diagram of the CDRS-R, with significant standardized factor loadings (*p* < 0.001). Although the correlations between factors in the four-factor model were high (r > 0.8), they were kept separate to maintain the original theoretical framework.

#### 3.3.3. ROC Analysis

**Figure 2:**
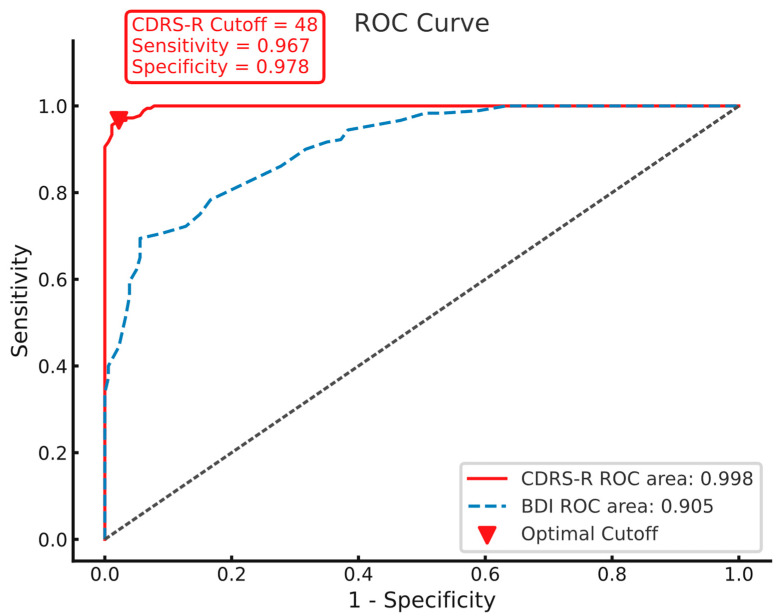
The ROC curve.

The ROC curve is shown in Figure 2. The ROC analysis indicated that a CDRS-R score of 48 served as the optimal cutoff value, with a sensitivity of 0.967 and a specificity of 0.978. An AUC of 0.998 (95% CI: 0.995–1.000) was obtained from the ROC analysis, which indicated that the CDRS-R had excellent diagnostic accuracy among Chinese adolescents. The AUC of CDRS-R demonstrated its ability to differentiate between MDD and HCs. The pairwise comparison of AUC values between the two scales revealed that the CDRS-R outperformed the BDI in diagnostic efficiency (*p* < 0.001).

#### 3.3.4. Analysis of Age and Gender Differences in CDRS-R Scores

In the full sample, Spearman’s correlation analysis revealed no significant relationship between age and CDRS-R scores. However, when analyzed separately significant negative correlations between age and CDRS-R scores were found in both the MDD group (r = −0.191, *p* = 0.010) and the HC group (r = −0.148, *p* = 0.047) (see Supplementary Table S3).

In the full sample, the Mann–Whitney U test analyses indicated no significant gender differences in CDRS-R scores. However, when the groups were analyzed separately significant gender differences emerged in both the MDD group (*p* = 0.001, Cohen’s d = 0.59) and HC group (*p* = 0.031, Cohen’s d = 0.27) (see Supplementary Table S4).

## 4. Discussion

This study aimed to assess the psychometric properties of the CDRS-R among Chinese adolescents, marking the first evaluation of its characteristics in China. The Cronbach’s alpha coefficient for the CDRS-R was 0.966, reflecting excellent reliability, consistent with previous findings where the coefficient ranged from 0.70 to 0.92 in different settings [15,17,24].

The convergent validity of the Chinese CDRS-R was supported by comparisons with other depression measures. The CDRS-R total score showed significant correlations with scores on the K-SADS-PL, BDI, and CGI-S, aligning with findings from previous studies [15,24]. The CDRS-R items, initially proposed by Poznanski et al., were grouped into four domains: mood, somatic, subjective, and behavioral [29]. Previous exploratory factor analyses have evaluated the dimensions of the scale, but the results have been inconsistent. Guo et al. identified a five-factor structure for the CDRS-R [17]; Kim et al., on the other hand, through an exploratory factor analysis, identified three factors for the Korean version of the CDRS-R: subjective depressed mood, impairment of daily functioning, and observed depressed mood [24]. However, these studies had some limitations, such as small sample sizes and repeated assessments of the same subjects using the same depression scales over relatively short time intervals (1–2 weeks), which may have led to bias due to testing effects [24]. The data were derived from multiple clinical trials, but the number of raters involved in data collection or their training during the study period could not be determined [17]. However, in our study both interviewers underwent comprehensive training in clinician rating scales before conducting the interviews. Each participant was administered the K-SADS-PL-C DSM-5 test, and depression symptom questions were asked repeatedly during the interview to ensure the accuracy of the CDRS-R score. The structural validity of the Chinese version of the CDRS-R was assessed through CFA. Our findings provided support for the original four-factor model, meeting satisfactory quality criteria in CFA. These results underscore the validity and applicability of the CDRS-R in the Chinese cultural context.

Our research also aimed to assess the diagnostic efficiency of the CDRS-R using ROC analysis and comparing the AUC values of the BDI and CDRS-R. The CDRS-R demonstrated a notably greater diagnostic efficiency, evidenced by a superior AUC in comparison to the BDI. This result is in agreement with earlier research conducted by Kim et al. [24]. The findings highlight the effectiveness of the CDRS-R in evaluating depressive symptoms in adolescents. This superiority may be attributed to its tailored design, comprehensive item coverage, and structured interview format, which facilitate a thorough evaluation of pediatric depression. Furthermore, the rigorous translation process of the Chinese version likely enhanced its cultural relevance and linguistic appropriateness, thus contributing to its diagnostic accuracy. In summary, our study underscores the importance of utilizing psychometrically validated instruments such as the CDRS-R for precise depression assessment in adolescents, which is crucial for both clinical practice and research endeavors.

### Limitations

While our study provided valuable insight into the reliability and validity of the Chinese version of the CDRS-R, it has several limitations that require consideration. First, the sample for this study was primarily drawn from a single center, which may have introduced sampling bias. Secondly, the analysis did not include the patient’s medication status, and because this study is cross-sectional, it was unable to assess the scale’s sensitivity to changes in treatment. Thirdly, the current sample was limited to adolescents with MDD and healthy controls, and the AUCs from this study might require cautious interpretation as the metrics used could have inflated the effect size [35]. Additionally, this study did not include test–retest reliability assessments, which limits the ability to verify the stability and consistency of the results over time. Future studies should consider incorporating test–retest reliability assessments to address this limitation and ensure more reliable findings.

## 5. Conclusions

We found that the CDRS-R demonstrates strong reliability and validity in diagnosing MDD among Chinese adolescents, rendering it an effective tool. Currently, there are no clinically validated tools for assessing depressive symptoms in adolescents in China. This study provides a reliable instrument for Chinese psychiatrists to diagnose and assess depressive symptoms in adolescents.

## Figures and Tables

**Table 1:** Characteristics of the participants.

Characteristics	MDD	HCs	*p*-Value	Cohen’s d
Patients (n)	180	180	-	
Female (n, %) ^a^	108, 60%	112, 62.2%	*p* = 0.665	0.02
Age ^b^	14.72 ± 1.62	14.51 ± 1.17	*p* = 0.664	0.15
BMI ^b^	20.48 ± 3.51	19.92 ± 3.01	*p* = 0.220	0.17
K-SADS-PL-C depression subscale ^b^	26.76 ± 2.43	7.42 ± 6.38	*p* < 0.001	4.01
CDRS-R ^b^	73.50 ± 13.59	26.48 ± 8.29	*p* < 0.001	4.18
BDI ^b^	30.72 ± 10.16	13.10 ± 8.39	*p* < 0.001	1.89
CGI-S ^b^	5.42 ± 0.72	1.88 ± 0.90	*p* < 0.001	4.35

Abbreviations: MDD, major depressive disorder; HCs, healthy controls; BMI, body mass index; CDRS-R, Children’s Depression Rating Scale—Revised; BDI, Beck Depression Inventory; K-SADS-PL-C, Chinese version of the Kiddie Schedule for Affective Disorders and Schizophrenia—Present and Lifetime Version; CGI-S, Clinical Global Impressions—Severity. Continuous variables are expressed as the mean ± standard deviation (SD). ^a^ Analyzed by the Chi-square test. ^b^ Analyzed by Mann–Whitney U test.

**Table 2:** Internal consistency and item–total correlations.

Factors/Items	Item–Total Correlation	Cronbach’s Alpha
Factor 1: Mood		0.882
Irritability	0.783 **	-
Depressed feelings	0.938 **	-
Excessive weeping	0.760 **	-
Depressed affect	0.870 **	-
Factor 2: Somatic		0.891
Sleep disturbance	0.782 **	-
Appetite disturbance	0.806 **	-
Excessive fatigue	0.882 **	-
Physical complaints	0.811 **	-
Listless speech	0.777 **	-
Hypoactivity	0.759 **	-
Factor 3: Subjective		0.848
Excessive guilt	0.836 **	-
Low self-esteem	0.821 **	-
Morbid ideation	0.642 **	-
Suicidal ideation	0.848 **	-
Factor 4: Behavior		0.910
Impaired schoolwork	0.892 **	-
Difficulty having fun	0.897 **	-
Social withdrawal	0.816 **	-
Total Cronbach’s alpha		0.966

** *p*-value < 0.01.

**Table 3:** Correlations between CDRS-R and other related assessments.

	CDRS-R	K-SADS-PL-C Depression Subscale	BDI	CGI-S
CDRS-R	1	-	-	-
K-SADS-PL-C depression subscale	0.886 **	1	-	-
BDI	0.871 **	0.789 **	1	-
CGI-S	0.937 **	0.868 **	0.857 **	1

** *p*-value < 0.01. Abbreviations: CDRS-R, Children’s Depression Rating Scale—Revised; BDI, Beck Depression Inventory; K-SADS-PL-C, Chinese version of the Kiddie Schedule for Affective Disorders and Schizophrenia—Present and Lifetime Version; CGI-S, Clinical Global Impressions—Severity.

## Data Availability

The data of this study are available from the corresponding author upon reasonable request.

## Supplementary Materials

The following supporting information can be downloaded at https://www.mdpi.com/article/10.3390/healthcare13070734/s1. Table S1: Correlations between CDRS-R and other related assessments in the MDD group; Table S2: Correlations between CDRS-R and other related assessments in the HC group; Table S3: Spearman’s Correlation Between Age and CDRS-R Scores; Table S4: Gender differences in CDRS-R Scores.

## Abbreviations

The following abbreviations are used in this manuscript:

CDRS-R	Children’s Depression Rating Scale—Revised
MDD	Major depressive disorder
HCs	Healthy controls
ROC	Receiver operating characteristic
BDI	Beck Depression Inventory
CES-DC	Center for Epidemiologic Studies Depression Scale for Children
HAMD	Hamilton Depression Rating Scale
K-SADS-PL-C	Chinese version of Kiddie Schedule for Affective Disorders and Schizophrenia for School-Age Children—Present and Lifetime Version
DSM-5	Diagnostic and Statistical Manual of Mental Disorders—Fifth Edition
CGI-S	Clinical Global Impressions—Severity
SDs	Standard deviations
CFA	Confirmatory factor analysis
MLR	Robust Maximum Likelihood
CFI	Comparative fit index
TLI	Tucker–Lewis index
SRMR	Standardized root mean square residual
RMSEA	Root mean square error of approximation
AUC	Area under the curve
