# Supplementary material for: Reliability and Validity of the Chinese Version of the Children’s Depression Rating Scale—Revised (CDRS-R)
Source: Healthcare (Basel). 2025 Mar 26;13(7):734. doi: 10.3390/healthcare13070734 (PMC11988789; doi:10.3390/healthcare13070734)
Supplement: Supplementary file 1 [file healthcare-13-00734-s001.zip › healthcare-3450580-supplementary.pdf]

Reliability and validity of the Chinese version of the Children's Depression Rating Scale—Revised (CDRS-R)

Supplementary Material

Supplementary Table S1. 2

Supplementary Table S2. 2

Supplementary Table S3. 3

Supplementary Table S4. 3

How to Obtain Authorization for the CDRS-R？ 4

Supplementary Table S1. Correlations between CDRS-R and other related assessments in the MDD group.

	CDRS-R	K-SADS-PL-C Depression subscale	BDI	CGI-S
CDRS-R	1	-	-	-
K-SADS-PL-C Depression subscale	0.710**	1	-	-
BDI	0.749**	0.554**	1	-
CGI-S	0.731**	0.469**	0.652**	1

** *p*-value < 0.01.

Abbreviations: CDRS-R, Children's Depression Rating Scale—Revised; BDI, Beck Depression Inventory; K-SADS-PL-C, Chinese version of the Kiddie Schedule for Affective Disorders and Schizophrenia—Present and Lifetime Version; CGI-S, Clinical Global Impressions—Severity.

Supplementary Table S2. Correlations between CDRS-R and other related assessments in the HC group.

	CDRS-R	K-SADS-PL-C Depression subscale	BDI	CGI-S
CDRS-R	1	-	-	-
K-SADS-PL-C Depression subscale	0.393**	1	-	-
BDI	0.747**	0.443**	1	-
CGI-S	0.746**	0.429**	0.781**	1

**P-value < 0.01.

Abbreviations: CDRS-R, Children's Depression Rating Scale—Revised; BDI, Beck Depression Inventory; K-SADS-PL-C, Chinese version of the Kiddie Schedule for Affective Disorders and Schizophrenia—Present and Lifetime Version; CGI-S, Clinical Global Impressions—Severity.

Supplementary Table S3. Spearman's Correlation Between Age and CDRS-R Scores.

**Group**	**Age (Mean±SD)**	**CDRS-R (Mean±SD)**	**r**	***p*-value**
Full Sample	14.61±1.41	49.99±26.09	-0.069	P = 0.189
MDD	14.72±1.62	73.50±13.59	-0.191	P = 0.010
HCs	14.51±1.17	26.48±8.29	-0.148	P = 0.047

Abbreviations: MDD, major depressive disorder; HCs, healthy controls; CDRS-R, Children's Depression Rating Scale—Revised; SD, standard deviation.

Supplementary Table S4. Gender differences in CDRS-R Scores.

**Group**	**Gender**	**Number**	**CDRS-R (Mean±SD)**	***p*-value**	**Cohen's d**
Full Sample	Male	140	47.62±24.62	P = 0.070	0.15
	Female	220	51.5±26.93		
MDD	Male	72	68.89±13.56	P = 0.001	0.59
	Female	108	76.57±12.78		
HCs	Male	68	25.10±7.91	P = 0.031	0.27
	Female	112	27.31±8.44		

Abbreviations: MDD, major depressive disorder; HCs, healthy controls; CDRS-R, Children's Depression Rating Scale—Revised; SD, standard deviation.

How to Obtain Authorization for the CDRS-R？

The Children's Depression Rating Scale—Revised (CDRS-R) is a copyrighted tool owned by Western Psychological Services (WPS). Researchers who wish to use the CDRS-R, including its Traditional Mandarin and Simplified Chinese translations, must obtain authorization from WPS.

Email: rights@wpspublish.com.

Website address：https://www.wpspublish.com/cdisc-collaborators-page
